# Vigilância e saúde pública nas fronteiras: uma análise dos eventos de notificação imediata no Paraná, Brasil (2010-2019)

**DOI:** 10.1590/0102-311XPT058525

**Published:** 2025-12-01

**Authors:** Daniele Akemi Arita, Vera Lúcia Machado Calliari, Aline Araújo Nobre, Claudia Torres Codeço

**Affiliations:** 1 Programa Educacional em Vigilância em Saúde nas Fronteiras, Fundação Oswaldo Cruz, Rio de Janeiro, Brasil.; 2 Centro de Informações Estratégicas de Vigilância em Saúde, Secretaria de Estado da Saúde do Paraná, Curitiba, Brasil.; 3 Programa de Computação Científica, Fundação Oswaldo Cruz, Rio de Janeiro, Brasil.

**Keywords:** Notificação de Doenças, Saúde na Fronteira, Estudos Transversais, Disease Notification, Border Health, Cross-Sectional Studies, Notificación de Enfermedades, Salud Fronteriza, Estudios Transversales

## Abstract

Fronteiras internacionais apresentam risco sanitário pela intensa circulação de pessoas e produtos, demandando vigilância e intervenções em saúde pública específicas. No Brasil, a diversidade territorial amplia esses desafios. O objetivo deste estudo é analisar o padrão de ocorrência de eventos de notificação imediata de saúde pública nas regiões de fronteira do Brasil e, mais especificamente, no Estado do Paraná, de 2010 a 2019. São analisados 14 eventos da Lista Nacional de Notificação Compulsória de Doenças, de notificação imediata para os três níveis governamentais, com dados do Departamento de Informação e Informática do SUS. Para cada evento, comparou-se a incidência entre municípios fronteiriços e não fronteiriços nos níveis de Brasil e Estado do Paraná. Calcularam-se frequências absolutas e relativas, incidência e taxa de mortalidade (por 100 mil habitantes). Para comparar o excesso de risco entre grupos, calculou-se razão de taxas (RT). Foram registrados 50.628 eventos de saúde pública no período, sendo mais frequentes casos de sarampo (66,44%) e óbitos de dengue (11,52%). Nas fronteiras, concentraram-se casos de malária extra-amazônica (RT = 32,41), botulismo (RT = 6,94), hantavirose (RT = 2,49) e óbitos por dengue (RT = 1,39). Destaca-se o sarampo, com menores incidências registradas nas regiões fronteiriças. A dinâmica das doenças malária extra-amazônica, dengue, hantavirose e botulismo em fronteiras foi diferenciada em relação ao restante do país. A alta incidência nas fronteiras expõe vulnerabilidades críticas, demandando ações urgentes e coordenadas. É imprescindível fortalecer a vigilância e promover estratégias intersetoriais, com cooperação, para enfrentar os desafios únicos dessas áreas. Fortalecer unidades estratégicas, como o Centro de Informações Estratégicas de Vigilância em Saúde, aprimora a vigilância, integração e garante respostas rápidas.

## Abstract (en)

International borders pose health risks due to the intense movement of people and products, requiring surveillance and specific public health interventions. In Brazil, territorial diversity accentuates these challenges. The aim of the present study was to analyze the pattern of the mandatory immediate reporting of public health events in border regions of Brazil and more specifically in the state of Paraná from 2010 to 2019. Fourteen events from the National List of Compulsory Notification of Diseases that require immediate reporting to the three levels of government were analyzed using data from the Brazilian Health Information and Informatics Department. For each event, incidence rates were compared between border and non-border municipalities on two levels (nationally and in the state of Paraná). Absolute and relative frequencies as well as incidence and mortality rates (per 100,000 inhabitants) were calculated. Rate ratios (RR) were calculated to compare excess risk between groups. A total of 50,628 public health events occurred in the period studied; the most frequent were cases of measles (66.44%) and deaths related to dengue (11.52%). Cases of extra-Amazonian malaria (RR = 32.41), botulism (RR = 6.94), hantavirus (RR = 2.49), and deaths related to dengue (RR = 1.39) were concentrated at the borders. The incidence of measles was lower in border regions. The dynamics of extra-Amazonian malaria, dengue, hantavirus, and botulism at borders differed from those in the rest of the country. The high incidence at borders reveals critical vulnerabilities that require urgent, coordinated action. It is essential to strengthen surveillance and promote shared intersectoral strategies to address the unique challenges of these areas. Strengthening strategic units, such as the Strategic Information Center for Health Surveillance, improves surveillance and integration and ensures rapid responses.

## Abstract (es)

Las fronteras entre países exponen riesgos para la salud debido a la intensa circulación de personas y productos, que requiere vigilancia específica e intervenciones de salud pública. En Brasil, la diversidad territorial intensifica aún más estos desafíos. El objetivo de este estudio es analizar el patrón de ocurrencia de eventos de notificación inmediata de salud pública en las regiones fronterizas de Brasil, y más específicamente en el estado de Paraná, para el período de 2010 a 2019. Se analizaron 14 eventos de la Lista Nacional de Enfermedades de Notificación Obligatoria, de notificación inmediata a los tres niveles gubernamentales, con datos del Departamento de Información e Informática del Sistema Único de Salud. Para cada evento, se comparó la incidencia entre los municipios fronterizos y no fronterizos a nivel de Brasil y el estado de Paraná. Se calcularon las frecuencias absolutas y relativas, la incidencia y la tasa de mortalidad (por cada 100.000 habitantes). Para comparar el exceso de riesgo entre grupos, se calculó la razón de tasas (RT). Hubo 50.628 eventos de salud pública en el período, y los más frecuentes fueron los casos de sarampión (66,44%) y las muertes por dengue (11,52%). En las fronteras se concentraron los casos de malaria extraamazónica (RT = 32,41), botulismo (RT = 6,94), infecciones por hantavirus (RT = 2,49) y muertes por dengue (RT = 1,39). Destaca el sarampión con menor incidencia registrada en las regiones fronterizas. Las dinámicas de las enfermedades malaria extraamazónica, dengue, infecciones por hantavirus y botulismo en las fronteras se diferenciaron con relación al resto del país. La alta incidencia de estos resultados expone vulnerabilidades críticas que requieren una acción urgente y coordinada. Es imprescindible fortalecer la vigilancia y promover estrategias intersectoriales, con cooperación, para abordar los desafíos únicos de estas áreas. El fortalecimiento de unidades estratégicas como el Centro de Información Estratégica de Vigilancia Sanitaria permite mejorar la vigilancia, la integración y garantiza respuestas rápidas.

## Introdução

Em um mundo globalizado, as fronteiras são estratégicas para conter doenças infecciosas e garantir segurança sanitária ^1^. Essas regiões representam desafios únicos à saúde pública ^2^, por concentrarem fluxos de pessoas, animais e mercadorias, potenciais vetores de riscos. Historicamente, surtos como Influenza A (H1N1) em 2009 ^3^, Ebola (2013-2016) ^4^ e SARS-CoV-2 ^5^ evidenciam essa vulnerabilidade.

O Brasil, com suas fronteiras continentais (10 países), apresenta três arcos fronteiriços distintos ^6^: o Arco Norte, com baixa densidade populacional, comunidades indígenas/ribeirinhas e fluxos transfronteiriços históricos; o Arco Central, com ocupação densa e pecuária extensiva; e o Arco Sul, dinâmico economicamente (MERCOSUL), destacando-se a tríplice fronteira Brasil-Paraguai-Argentina ^7^. Essas regiões são estratégicas para vigilância em saúde pública, devido ao intenso fluxo internacional, diversidade ecológica, desigualdades econômicas, histórico de surtos e importância geopolítica ^6^, constituindo portas de entrada para doenças globais.

As fronteiras demandam estratégias diferenciadas de controle de doenças em comparação com outras áreas nacionais ^8^. No Brasil, a complexidade territorial amplia esse desafio, exigindo tanto a vigilância de agravos transfronteiriços quanto a caracterização daqueles endêmicos nessas regiões. Exemplificam essa particularidade os perfis distintos de tuberculose ^9^, malária ^10^ e arboviroses ^11^ observados nas fronteiras, que divergem das taxas nacionais.

O Regulamento Sanitário Internacional (2005) ^12^ exige que os países mantenham capacidades básicas de saúde pública em pontos de entrada, como aeroportos e fronteiras. O Brasil, como signatário, deve compartilhar informações epidemiológicas mediante solicitação oficial do ponto focal de outros países ou da Organização Mundial da Saúde (OMS), assegurando coordenação nas ações de vigilância global. Em maio de 2024, o país instituiu a Política Nacional de Fronteiras e seu Comitê Nacional ^13^, focados na prevenção, vigilância e promoção da saúde por meio da cooperação interinstitucional.

Visando contribuir com a vigilância de fronteiras e atender a necessidade de estudos epidemiológicos que foquem nestes territórios, o objetivo deste estudo foi analisar o padrão de ocorrência de doenças, agravos e eventos de saúde pública de notificação imediata nas regiões de fronteira do Brasil e, mais especificamente, no Estado do Paraná, no período de 2010 a 2019. O foco nesse estado se justifica pela sua importância na composição da tríplice fronteira Brasil-Paraguai-Argentina, enquanto local de grande fluxo de pessoas e produtos. Esta análise fornecerá evidências para compreender a dinâmica espaço-temporal da notificação compulsória em áreas fronteiriças, visando sinalizar prioridades para intervenção e adequação dos protocolos de vigilância às realidades locais. Optou-se pelo estudo das doenças, agravos e eventos de notificação imediata por se tratar de condições que, devido à sua gravidade, alta transmissibilidade ou impacto potencial, exigem notificação imediata às autoridades de saúde.

A Lista Nacional de Notificação Compulsória de Doenças, Agravos e Eventos de Saúde Pública ^14^, regulamentada pelo Ministério da Saúde, define as doenças, agravos e eventos de saúde pública de notificação obrigatória em todo o território brasileiro, servindo como base para o sistema nacional de vigilância epidemiológica e, consequentemente, para esse estudo.

## Metodologia

O Brasil tem uma população de 203.080.756 habitantes ^15^, distribuídos em 5.570 municípios. Possui 588 municípios na faixa de fronteira, com uma população residente nesta área de 11.749.511 habitantes ^15^. A faixa de fronteira é definida como a região que dista até 150km da fronteira terrestre brasileira ^16^. O Paraná, área de interesse específica desse estudo, está localizado no Arco Sul, fazendo fronteira com Paraguai e Argentina. Dos 399 municípios desse estado, 139 (34,84%) são da faixa fronteiriça, com uma população residente nesta área de 2.672.357 habitantes (23,35%), de um total de 11.444.380 ^15^. O Paraná apresenta bons indicadores de saúde, com taxa de mortalidade infantil de 9,3 contra 11,20 do Brasil; expectativa de vida aos 60 anos de 23,5 anos contra 23,0 do Brasil; e índice de Gini de 0,47 contra 0,518 do Brasil ^17^.

O estudo ecológico descritivo foi realizado com dados agregados em escala municipal e anual, estratificado em região fronteiriça e não fronteiriça. No primeiro recorte, foram analisados todos os municípios brasileiros e, no segundo, apenas o Estado do Paraná, também estratificado em municípios fronteiriços e não fronteiriços.

O período do estudo foi de janeiro de 2010 a dezembro de 2019. O ano de 2020 marca o período inicial da pandemia de COVID-19, e a análise foi interrompida nesse momento devido aos potenciais impactos e distorções causados pelo contexto excepcional da crise sanitária.

### Eventos do estudo

Foram considerados os eventos de notificação imediata (em até 24 horas) para os três níveis governamentais e que apresentassem dados disponíveis no Departamento de Informação e Informática do SUS (DATASUS; https://datasus.saude.gov.br/). Para fins deste estudo, adotou-se o termo “eventos” como forma simplificada de se referir ao conceito amplo de “doenças, agravos e eventos de saúde pública”, mantendo-se essa padronização terminológica em todo o documento.

Dos 62 eventos da Lista Nacional de Notificação Compulsória de Doenças, Agravos e Eventos de Saúde Pública ^14^, 29 são de notificação imediata para os três níveis de governo. Destes, 14 apresentam dados disponíveis no DATASUS: casos de raiva humana, paralisia flácida aguda, peste, malária extra-amazônica, hantavirose, febre maculosa, febre amarela, cólera, botulismo, sarampo, rubéola, rubéola congênita, e óbitos por Zika e dengue. A definição de caso que resulta em notificação compulsória difere entre os agravos, refletindo suas especificidades epidemiológicas.

Apenas casos extra-amazônicos de malária são considerados de notificação compulsória de notificação imediata. Segundo dados do Ministério da Saúde ^18^, 99,9% da transmissão da malária no Brasil ocorre na Região Amazônica (notificados no Sistema de Informação de Vigilância Epidemiológica - SIVEP). Este estudo concentrou-se nos 0,1% que ocorrem na região extra-amazônica (notificados no Sistema de Informação de Agravos de Notificação - SINAN), e que, no Brasil, estão associados principalmente à transmissão residual na região de Mata Atlântica. Para Zika e dengue, óbitos são eventos de notificação imediata enquanto, para os demais agravos, a ocorrência do caso confirmado é o evento de notificação.

Os dados foram obtidos do SINAN via TabNet/DATASUS ^19^, com contagem anual de casos por agravo. Para a maioria dos agravos, utilizou-se o município de residência; já para hantavirose, febre maculosa, febre amarela e malária, adotou-se o município provável de infecção, por refletir melhor a dinâmica de transmissão vinculada a fatores ambientais e ecológicos específicos.

### Análise

Para comparar a ocorrência dos 14 eventos entre região de fronteira e não fronteira do Brasil, e do Paraná em particular, os municípios foram alocados em quatro grupos: Grupo 1 (municípios fronteiriços do Paraná, n = 139), Grupo 2 (municípios não fronteiriços do Paraná, n = 260), Grupo 3 (municípios fronteiriços do Brasil, n = 588) e Grupo 4 (municípios não fronteiriços do Brasil, n = 4.982).

Para comparar a ocorrência dos eventos entre anos e grupos de municípios, foram calculadas frequências absolutas e relativas de cada agravo. Indicadores de incidência e mortalidade foram calculados por 100 mil habitantes, considerando no denominador a população de 2015 ^20^, correspondente ao ponto médio do período.

Para medir o excesso de risco em municípios fronteiriços em relação aos não fronteiriços, utilizou-se a razão de taxas (RT). Para este cálculo, considerou-se no numerador a incidência ou mortalidade no grupo fronteiriço e, no denominador, a incidência ou mortalidade no grupo não fronteiriço. Para avaliar as diferenças entre os grupos, foi utilizado o teste de taxa de Poisson, adotando-se um nível de significância de 5%. Posteriormente, para os eventos mais prevalentes, foram construídos gráficos de série temporal, assim como mapas.

Os dados foram analisados no EpiInfo, versão 7.2 (https://www.cdc.gov/epiinfo/index.html), sendo utilizado o Microsoft Office Excel 2010 (https://products.office.com/) para elaboração de tabelas e gráficos e o QGIS, versão 3.28.0 (https://qgis.org/en/site/), para mapas.

### Aspectos éticos

O estudo foi aprovado no Comitê de Ética em Pesquisa da Escola Nacional de Saúde Pública Sergio Arouca, Fundação Oswaldo Cruz, em 20 de março de 2024 (parecer nº 6.713.232).

## Resultados

No período, foram registrados no Brasil 50.628 eventos de saúde pública, dentre os 14 agravos analisados nesse estudo. Em ordem decrescente: 33.639 casos de sarampo (66,44% do total), 5.832 óbitos de dengue (11,52%), 5.031 casos de paralisia flácida aguda (9,94%), 2.278 de febre amarela (4,50%), 1.364 de febre maculosa (2,69%), 1.113 de malária extra-amazônica (2,20%), 965 de hantavirose (1,91%), 196 de rubéola (0,39%), 75 óbitos de Zika (0,15%), 61 casos de botulismo (0,12%), 34 de rubéola congênita (0,07%), 37 de raiva humana (0,07%) e três de cólera (0,01%). Nenhum caso de peste foi notificado.

**Figura 1:**
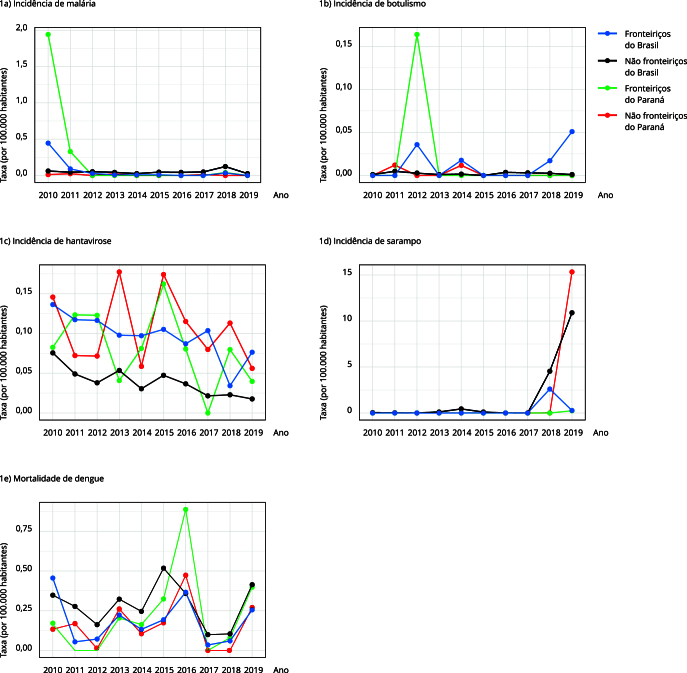
Séries temporais de taxas de incidência de doenças de notificação obrigatória em faixa de fronteira e área de não fronteira do Paraná, em todo o Brasil (2010-2019).

A Tabela 1 apresenta as incidências e mortalidades por grupo de municípios. Dos 14 agravos, seis apresentaram taxas de incidências distintas na região de fronteira em comparação com o restante dos municípios. Hantavirose e botulismo apresentaram maior incidência na região de fronteira brasileira, enquanto febre maculosa, febre amarela, sarampo e rubéola apresentaram significativamente menor incidência nessa região. A mortalidade por dengue também foi significativamente menor nessa região. No caso específico do Paraná, apenas três agravos apresentaram diferenças entre as áreas fronteiriças e não fronteiriças: malária, dengue e sarampo. Assim como no restante do país, verificou-se menor incidência de sarampo na fronteira do Paraná. Em contrapartida, a mortalidade por dengue foi significativamente maior na fronteira em relação aos demais municípios do estado. Padrão semelhante foi observado para a malária extra-amazônica, embora esteja restrita ao início do período (Figura 1).

**Tabela 1:** Taxa de incidência, mortalidade (por 100 mil habitantes) e razão de taxas (RT) dos eventos de notificação imediata a todas as esferas de governo por grupos de municípios fronteiriços e não fronteiriços, Paraná e Brasil (2010-2019).

Eventos	Grupo 1	Grupo 2	1/2	Grupo 3	Grupo 4	3/4	Total
	Fronteiriços do Paraná	Não fronteiriços do Paraná	RT	Fronteiriços do Brasil	Não fronteiriços do Brasil	RT	
Incidência							
Raiva humana	0,00	0,001	0,00	0,004	0,002	2,10	0,002
Paralisia flácida aguda	0,30	0,24	1,26	0,26	0,23	1,11	0,23
Peste	0,00	0,00	-	0,00	0,00	-	0,00
Malária extra-amazônica	0,23	0,01	32,41 *	0,06	0,05	1,18	0,05
Hantavirose	0,08	0,11	0,76	0,10	0,04	2,49 *	0,04
Febre maculosa	0,05	0,04	1,19	0,02	0,07	0,27 *	0,07
Febre amarela	0,00	0,01	0,00	0,01	0,12	0,04 *	0,11
Cólera	0,00	0,00	-	0,00	0,0002	0	< 0,001
Botulismo	0,02	0,002	6,94	0,01	0,002	5,74 *	0,003
Sarampo	0,02	1,60	0,02 *	0,30	1,67	0,18 *	1,59
Rubéola	0,00	0,002	0,00	0,003	0,01	0,27 *	0,01
Rubéola congênita	0,00	0,00	-	0,0009	0,002	0,51	0,002
Mortalidade							
Zika	0,00	0,00	-	0,0052	0,0074	0,70	0,01
Dengue	0,22	0,16	1,39 *	0,18	0,28	0,65 *	0,40

* Valor de p < 0,05.

### Distribuição temporal e espacial de eventos com maior risco na fronteira

**Figura 2:**
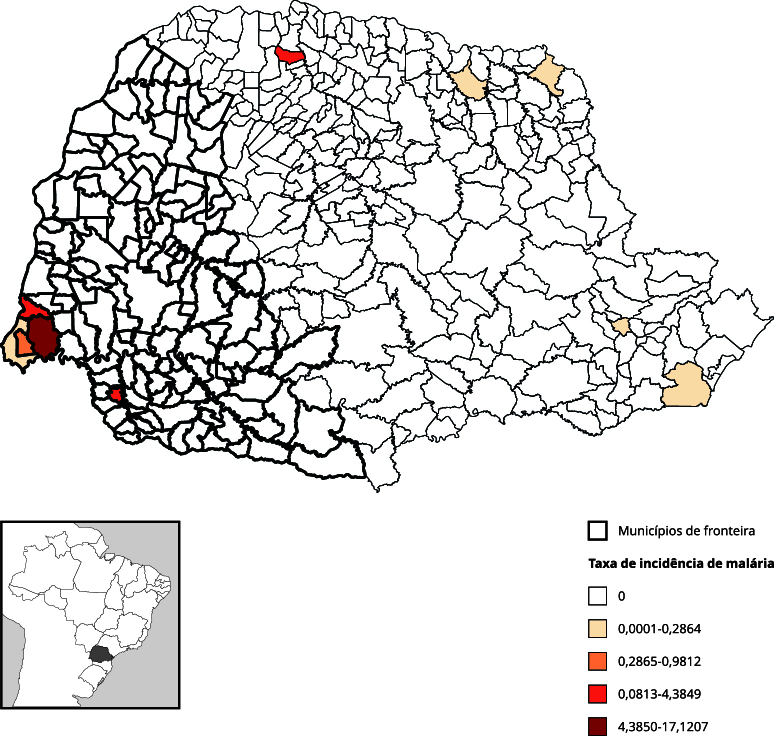
Mapa de taxa de incidência média de malária extra-amazônica por 100 mil habitantes. Paraná, Brasil (2010-2019).

A Figura 1 mostra a distribuição temporal dos eventos que apresentaram maior risco na fronteira, seja no Brasil em geral ou, especificamente, no Paraná. A malária extra-amazônica é um caso particular, pois a análise está restrita aos Arcos Sul e Central (extra-amazônicos). No seu período de maior incidência na fronteira do Paraná, 2010-2011, a malária apresentava-se concentrada na região da tríplice fronteira (Figura 2), sem disseminação para o restante do estado.

**Figura 3:**
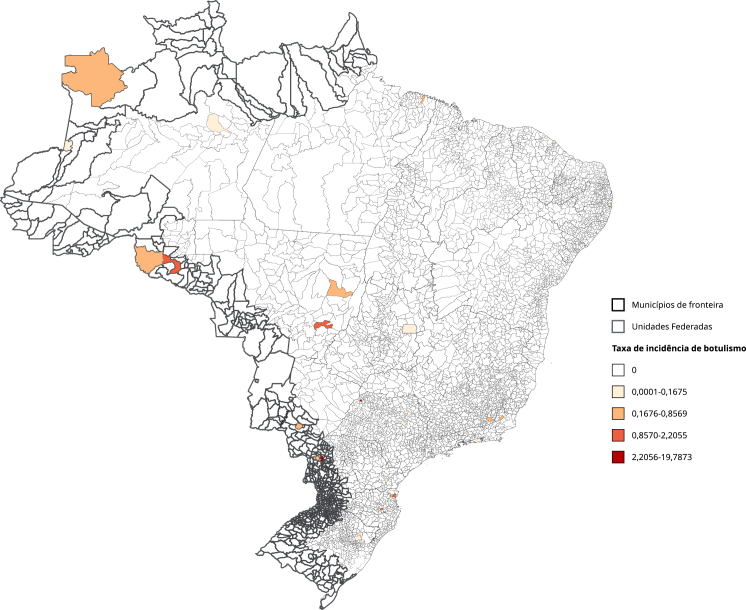
Mapa de taxa de incidência média de botulismo por 100 mil habitantes. Brasil (2010-2019).

O botulismo, por sua vez, apresentou picos de incidência em 2012, 2014 e ao final do período, 2018 e 2019 (Figura 1). O maior risco concentrou-se na região de Rondônia, no Arco Norte da fronteira brasileira (Figura 3). Apesar dos valores altos de RT, observa-se uma baixa incidência no período analisado (Tabela 1).

**Figura 4:**
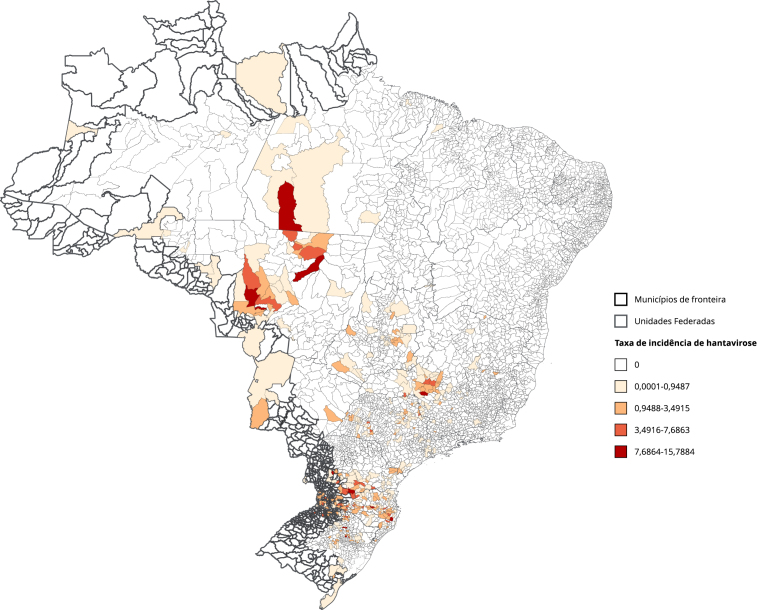
Mapa de taxa de incidência média de hantavirose por 100 mil habitantes. Brasil (2010-2019).

A hantavirose apresentou maior risco no Arco Central da fronteira brasileira, com concentração adicional de casos em áreas não fronteiriças, especialmente nos estados do Mato Grosso e Sul do Pará, todas áreas com grande ocupação agrícola (Figura 4). Embora notificada todos os anos, apresenta aparente queda nas taxas ao longo do período, especialmente após 2018, em todo o país (Figura 1).

**Figura 5:**
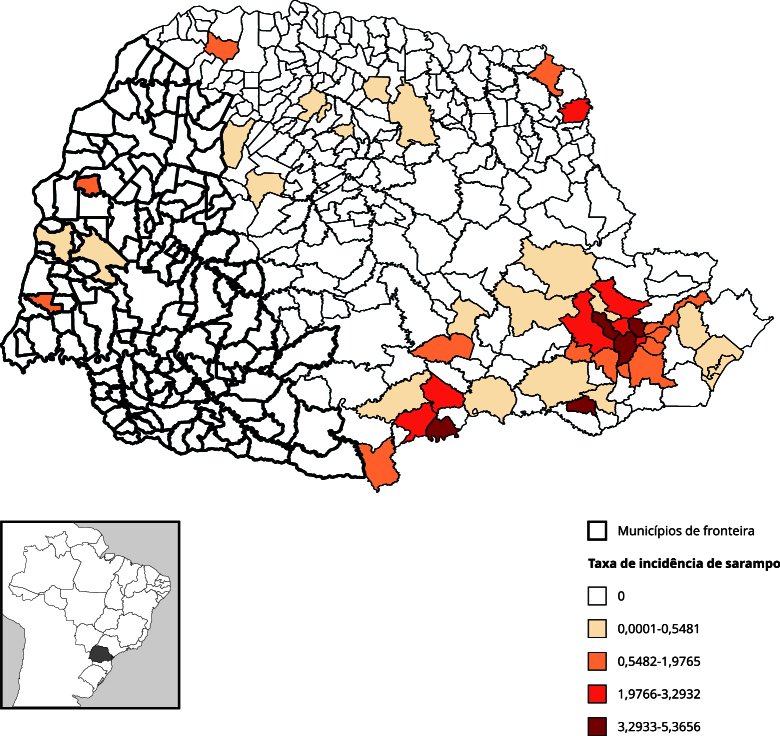
Mapa de taxa de incidência média de sarampo por 100 mil habitantes. Paraná, Brasil (2010-2019).

**Figura 6:**
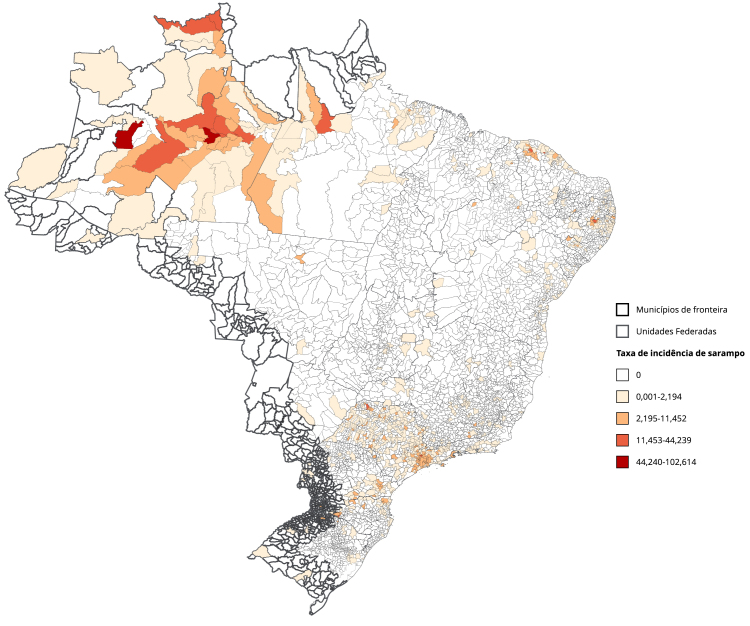
Mapa de taxa de incidência média de sarampo por 100 mil habitantes. Brasil (2010-2019).

De 2010 a 2017, o sarampo apresentou baixas incidências no Brasil (Figura 1). No entanto, a partir de 2018 observou-se uma alteração importante no comportamento epidemiológico da doença, com a reintrodução do vírus a partir de casos importados de outros países com circulação ativa do sarampo. Houve o ressurgimento de surtos e o aumento das taxas de incidência (Figura 1), especialmente em regiões não fronteiriças do Paraná (Figura 5) e de outras áreas do Brasil (Figura 6).

**Figura 7:**
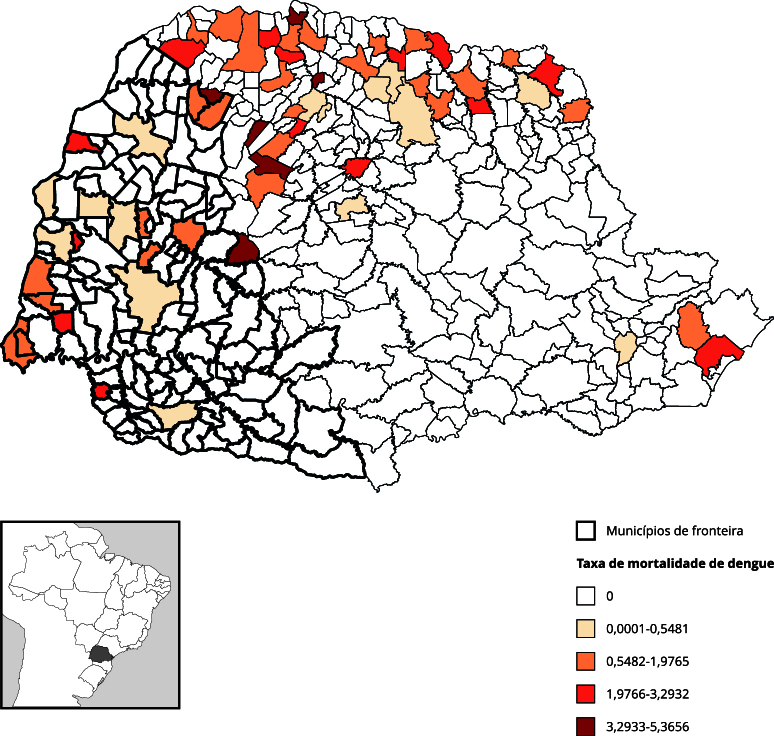
Mapa de taxa de mortalidade média de dengue por 100 mil habitantes. Paraná, Brasil (2010-2019).

As taxas de mortalidade por dengue permaneceram baixas até atingirem um pico em 2016 (Figura 1), superando a série histórica analisada e com importante tendência de acréscimo. No Paraná, a mortalidade foi maior em áreas de fronteira, ao contrário do observado no Brasil, onde o risco foi mais elevado fora da faixa fronteiriça. No estado, óbitos por dengue predominaram na região noroeste, que inclui a fronteira (Figura 7). Esses aumentos coincidem com períodos epidêmicos da dengue no país.

## Discussão

Nas últimas décadas, mudanças socioeconômicas e ambientais alteraram hábitos e dinamizaram o fluxo migratório global, facilitando a disseminação de doenças. Agravadas por alterações climáticas, essas transformações aumentaram o risco de epidemias por patógenos emergentes oriundos da vida selvagem, tornando-se uma ameaça mundial ^21^. Este estudo analisou eventos de saúde relevantes nas fronteiras do Brasil e do Paraná. No período estudado, os agravos de maior risco foram malária extra-amazônica e óbitos por dengue (fronteira do Paraná), botulismo e hantavirose (faixa de fronteira do Brasil). Já o sarampo apresentou maior incidência em regiões não fronteiriças, tanto nacionalmente quanto no Paraná.

A alta ocorrência de malária na tríplice fronteira do Paraná, no início dos anos 2010, pode ser atribuída a alterações ambientais decorrentes da construção da usina hidrelétrica de Itaipu. Tais modificações ecológicas criaram condições favoráveis à proliferação de vetores, com aumento da densidade de populações específicas, como anofelinos ^22^. Esse fator, associado à ampla circulação de pessoas na fronteira resultaram em dezenas de casos importados e autóctones, sendo provável que alguns casos autóctones tenham sido secundários a casos importados ^23^. Adicionalmente, a pressão contínua pelo qual foi submetida a Mata Atlântica, resultante do impacto das atividades humanas, pode também ter contribuído no aumento dos casos ^24^. Entretanto, a importante redução na incidência, pode ser reflexo da intensificação do processo de eliminação da malária, adotado pelos países das Américas, incluindo o Paraguai, país vizinho do Estado do Paraná. Entre 2000 e 2015, o Brasil apresentou uma redução no número de casos de malária, atingindo um dos Objetivos de Desenvolvimento do Milênio ^18^. A OMS lidera esforços para eliminar a malária em 35 países até 2030 ^25^. Reforçar a vigilância em áreas indenes de fronteira é essencial para alcançar essa meta. Além disso, a letalidade por malária em região extra-amazônica tende a ser aproximadamente 50 vezes maior em comparação com a Região Amazônica ^26^. Esse fenômeno decorre principalmente da baixa familiaridade dos profissionais com a doença em áreas não endêmicas, somada a atrasos no diagnóstico e tratamento. Na Amazônia, a experiência clínica facilita o reconhecimento precoce e reduz óbitos, enquanto nas regiões extra-amazônicas a ocorrência esporádica favorece erros diagnósticos, subnotificação e pior prognóstico ^27^ ^,^ ^28^. Essa disparidade reforça a necessidade de capacitação contínua dos profissionais de saúde e de estratégias de vigilância epidemiológica adaptadas às realidades locais, visando à redução da morbimortalidade por malária em todo o território nacional.

Ao contrário das demais doenças, para as quais o evento de notificação é o caso confirmado, para a dengue, o evento de interesse é o óbito. A dengue é uma doença endêmica no país desde 1986, com epidemias sazonais. Apesar da alta incidência, a letalidade é baixa por ser uma doença de curso benigno na maioria dos casos. Contudo, casos graves podem evoluir rapidamente para óbito na ausência de diagnóstico oportuno e tratamento adequado ^29^. Assim, o óbito é um evento de alta importância para a vigilância. A maior taxa de mortalidade por dengue no Paraná pode refletir tanto maior incidência quanto falhas no manejo clínico ^29^ ^,^ ^30^ ^,^ ^31^. Os óbitos acompanham a distribuição espacial da doença, com transmissão persistente no noroeste do estado, favorecida pelo clima subtropical úmido e fatores populacionais ^32^. Variáveis climáticas (temperatura e pluviosidade) são preditoras da dengue em Foz do Iguaçu ^33^ ^,^ ^34^ ^,^ ^35^, e sua incorporação aos sistemas de vigilância pode aprimorar a predição de epidemias ^36^. Estudos locais ^37^ demonstram que o monitoramento de formas imaturas do vetor e a integração de dados entomológicos, epidemiológicos e entomovirológicos permitem a detecção precoce de áreas de risco e funcionam como alerta para transmissão de arboviroses.

O botulismo configura emergência em saúde pública devido à gravidade de seu quadro clínico e ao seu potencial epidêmico por transmissão alimentar ^38^, particularmente em regiões fronteiriças onde os desafios de vigilância são ampliados ^39^. A contaminação de produtos de amplo consumo pode gerar surtos expressivos, excedendo rapidamente a capacidade assistencial local ^38^. A introdução de alimentos atípicos demanda vigilância ativa combinada com capacitação permanente de profissionais para identificação rápida de ameaças, fortalecendo a segurança alimentar nessas regiões vulneráveis ^39^. No período do nosso estudo o botulismo apresentou baixa incidência; contudo, sua elevada letalidade e potencial epidêmico em regiões fronteiriças mantêm seu *status* prioritário para vigilância em saúde. A natureza esporádica deste evento não corrobora estimativa de risco, pois mesmo casos isolados podem desencadear surtos localizados e sobrecarregar o sistema de saúde. A vigilância na fronteira pode evitar que esses produtos entrem no país sem controle, garantindo a segurança alimentar ^40^. A vigilância do botulismo é indispensável para identificar rapidamente qualquer caso, evitar a disseminação da toxina e proteger a saúde pública. Mesmo sendo rara, a doença demanda uma resposta robusta e coordenada, sendo componente essencial da segurança alimentar e da saúde coletiva.

A hantavirose, zoonose emergente de alta letalidade ^41^ ^,^ ^42^, apresenta maior ocorrência em áreas rurais e silvestres fronteiriças, onde roedores reservatórios e variantes virais transnacionais ^43^ ^,^ ^44^ coexistem com fatores ecológicos e antropogênicos ^41^. Apesar de distribuída nacionalmente, concentra-se nas regiões Sul e Sudeste ^41^ ^,^ ^42^, com mais da metade dos casos em estados fronteiriços ^42^. A ausência de tratamento específico reforça a necessidade de diagnóstico precoce e suporte terapêutico adequado ^41^.

O sarampo foi o agravo mais notificado no período de estudo, com uma incidência menor na fronteira em relação ao restante do país. No ano de 2016 o sarampo foi declarado como eliminado do país, *status* alcançado pelas altas coberturas vacinais. Contudo, a mudança de *status* e alta incidência observada em seguida pode ser resultado da menor cobertura vacinal ^45^ ^,^ ^46^. Em 2018, um surto iniciado no Norte do Brasil, importado da Venezuela, culminou na ocorrência de casos que se disseminaram por vários estados, mostrando a importância da vigilância na fronteira. A reemergência de doenças imunopreveníveis em contextos de redução de cobertura vacinal vem sendo registrada em vários países ^47^ ^,^ ^48^. As baixas incidências nas regiões de fronteira podem estar relacionadas a estratégias direcionadas de busca ativa por indivíduos não vacinados e identificação de bolsões populacionais com baixa imunização ^49^, adicionalmente ao fato de que a propagação da doença ocorra mais rapidamente nos grandes centros urbanos das regiões extrafronteiras. O Brasil, com sua extensa faixa fronteiriça, oportuniza a implementação de ações conjuntas de vacinação. Nesse sentido, os gestores da saúde do Brasil e dos países que fazem fronteira envidam esforços contínuos de cooperação mútua, no intuito de fortalecer as atividades desempenhadas pelo MERCOSUL, na formulação de políticas de gestão e educação na saúde ^49^.

Diversos agravos mostraram incidência equivalente em regiões fronteiriças e não fronteiriças. A raiva humana no Brasil declinou entre 2006 e 2017, com maior concentração nas regiões Norte/Nordeste, especialmente casos associados a morcegos ^50^. Nas fronteiras, a vigilância é crucial para detecção precoce de doenças em humanos e animais, prevenindo a propagação de patologias como raiva, malária e febre amarela em áreas anteriormente controladas ^40^.

A epidemia de Zika no Brasil ocorreu entre 2015 e 2016, sendo um evento curto e intenso, mais prevalente no Nordeste brasileiro ^51^. No Paraná, com poucos casos registrados, a ausência de óbitos por esse agravo sugere a efetividade das ações de vigilância, prevenção e manejo clínico. Da mesma forma, a inexistência de casos de rubéola e rubéola congênita provavelmente reflete o êxito das estratégias locais, aliado à baixa letalidade dessas doenças. Esses resultados ressaltam a necessidade de manter sistemas de vigilância eficazes e ações contínuas de prevenção, essenciais para reduzir a morbimortalidade.

A ausência de casos humanos de febre amarela nos municípios fronteiriços do Paraná pode ser atribuída à intensificação vacinal no estado, especialmente em 2007 e 2008, nas áreas recomendadas, que incluem regiões de fronteira ^52^ ^,^ ^53^. O surto de 2018 no Sul, após o surto nacional de 2017, levou à ampliação da vacinação para alcançar uma cobertura mínima de 95% ^54^. Modelos de favorabilidade, baseados em dados de 2020 a 2024, indicam que Santa Catarina, Rio Grande do Sul, Paraná, Distrito Federal, Goiás, São Paulo, Mato Grosso do Sul e Minas Gerais reúnem as condições mais favoráveis à febre amarela, com corredores ecológicos principalmente em áreas não fronteiriças ^55^.

A vigilância de paralisia flácida aguda, sentinela para identificação oportuna da circulação do vírus da poliomielite, estabelece como meta, para cada estado do país, a coleta sistemática anual de uma amostra de fezes para cada 100 mil habitantes menores de 15 anos. Os achados de maiores incidências nas regiões fronteiriças denotam maior sensibilidade dos municípios nessas áreas, considerando a circulação do vírus da poliomielite em outros países e o risco de sua reintrodução no território brasileiro. Nesse contexto, nas regiões fronteiriças do nordeste africano, desde 1996, ocorrem reuniões para melhorar esta imunização, visando melhorar a colaboração entre a saúde e as autoridades das regiões transfronteiriças por meio da intensificação da sensibilidade da vigilância da paralisia flácida aguda para a erradicação da poliomielite ^56^. No Brasil, a avaliação da vigilância laboratorial da paralisia flácida aguda (2005-2014) demonstrou desempenho satisfatório em todas as regiões, reforçando a necessidade de manter a vigilância ativa aliada a altas coberturas vacinais ^57^.

Algumas limitações do estudo incluem a falta de dados de alguns eventos com vigilância no Brasil, registrados em sistemas diferentes do SINAN e, portanto, não disponíveis no DATASUS, o que pode ter levado a subestimações ou superestimações na análise. Além disso, a ausência de dados sobre doenças febris hemorrágicas emergentes, suspeitas de disseminação intencional, febre do Nilo Ocidental e poliomielite por poliovírus selvagem na base pública decorre da inexistência de sistemas de informação específicos, já que esses eventos são extraordinários e não circulam no país. Adicionalmente, alguns agravos possuem notificação restrita ao nível municipal, a exemplo de acidente por animal peçonhento e leptospirose, cuja comparação com dados estaduais do Paraná pode enfrentar limitações metodológicas, como subnotificação desigual, diferenças na temporalidade dos registros e disparidades na cobertura geográfica. Essas limitações somam-se às fragilidades inerentes aos sistemas de domínio público como o DATASUS (incompletude e subnotificação). Diante deste cenário, a integração dos sistemas de vigilância emerge como estratégia prioritária, demandando desenvolvimento de arquitetura modular interoperável, que preserve os bancos existentes enquanto supera a atual fragmentação.

Superando as limitações do sistema nacional de notificação, um sistema transfronteiriço integrado ofereceria vantagens estratégicas para a vigilância epidemiológica em fronteiras, desde que contemplasse padronização de protocolos, interoperabilidade tecnológica e governança multinacional, elementos críticos para potencializar a detecção precoce e a resposta coordenada em contextos de vulnerabilidade sanitária compartilhada.

## Conclusão

As regiões de fronteira apresentaram taxas distintas de malária extra-amazônica, dengue, hantavirose e botulismo em comparação com outras áreas, devido a fatores como clima, fluxo migratório, infraestrutura sanitária precária e exposição ambiental. Essas particularidades são potencializadas pela alta mobilidade populacional, exigindo vigilância adaptada e estratégias locais para controle eficaz. Essa mobilidade favorece a disseminação de doenças, impactando comunidades vizinhas ^58^. A cooperação transfronteiriça é vital para enfrentar crises de saúde de forma coordenada. A literatura evidencia que acordos intergovernamentais entre países fronteiriços favorecem a construção colaborativa de políticas de vigilância em saúde para regiões de fronteira ^59^. Entretando, essa cooperação enfrenta desafios importantes como a falta de diretrizes claras sobre competências institucionais; escassez de recursos humanos e materiais; e limitações no financiamento regional sustentável ^60^. Como resposta, gestores fronteiriços implementam estratégias de integração operacional entre municípios limítrofes, com foco específico na notificação oportuna de eventos epidemiológicos transfronteiriços. Ações como essas tentam estreitar as relações entre os serviços de saúde dos países e abrem precedentes para acordos formais de integração político-sanitária ^59^. Diante desse cenário desafiador, destaca-se a importância crítica da vigilância fronteiriça integrada para monitorar e prevenir a propagação de doenças infecciosas, por meio da detecção e resposta rápida a surtos, garantindo melhor preparação para futuras ameaças à saúde pública ^61^.

A dinâmica das doenças ao longo do tempo nas regiões de fronteira reflete a complexidade da transmissão, influenciada por fatores como sazonalidade, migração, mudanças ambientais e intervenções em saúde pública. Taxas de incidência elevadas em fases iniciais de surtos podem estar associadas, em parte, a níveis insuficientes de imunidade populacional. Observa-se que a implementação oportuna de medidas de controle tende a resultar em redução progressiva dos casos notificados, embora esse padrão possa variar conforme características específicas do agente etiológico, da população e das intervenções empregadas. No entanto, novos surtos podem surgir com mudanças ambientais ou sociais. Assim, a vigilância é essencial para ajustar políticas de saúde de acordo com cada contexto.

Neste sentido, o Centro de Informações Estratégicas de Vigilância em Saúde (CIEVS) é uma estrutura técnica vinculada ao Ministério da Saúde, criada para integrar e analisar dados epidemiológicos em tempo real, com o objetivo de antecipar e mitigar riscos à saúde pública. Em regiões de fronteira, sua atuação é essencial, pois monitora ameaças à saúde, agiliza a detecção e resposta a surtos, contribui para a cooperação internacional em parceria com o município e coordena ações de prevenção, fortalecendo a segurança sanitária dessas regiões.

A vigilância fronteiriça é essencial para conter ameaças como COVID-19, Ebola, malária e zoonoses, exigindo políticas públicas coordenadas entre governos, organizações internacionais e comunidades locais. Campanhas de vacinação eficazes (sarampo, poliomielite, febre amarela) protegem populações vulneráveis e preservam a saúde global ^61^. Essa integração estratégica fortalece o controle de surtos e agiliza respostas a emergências sanitárias transnacionais.

Para fortalecer a vigilância integrada, recomenda-se a implementação de sistemas de monitoramento bi/trinacional para malária, dengue, hantavirose e botulismo, com compartilhamento de dados em tempo real e padronização de protocolos de diagnóstico entre países, garantindo detecção oportuna e resposta rápida. É essencial estabelecer comitês binacionais para coordenar respostas a surtos e mapear rotas migratórias críticas, além de capacitar continuamente profissionais de saúde em diagnóstico diferencial (especialmente para dengue e botulismo) e realizar campanhas educativas para populações móveis sobre práticas alimentares seguras e reconhecimento de sintomas.

Recomenda-se fortalecer unidades como o CIEVS nas fronteiras, ampliar a colaboração internacional, monitorar continuamente para resposta rápida a emergências e desenvolver estudos temporais para embasar decisões.

## Data Availability

As fontes de informação utilizadas no estudo estão indicadas no corpo do artigo.
